# Impact of Biomedical Optics and Planning our Revised Scope

**DOI:** 10.1117/1.JBO.25.5.050101

**Published:** 2020-05-01

**Authors:** Brian W. Pogue

## Abstract

Journal of Biomedical Optics editor-in-chief Brian W. Pogue outlines a revision of scope for the journal.

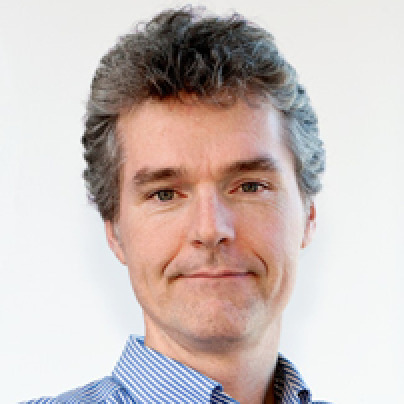



The field of biomedical optics has evolved over many decades, where devices and methods now penetrate nearly every aspect of medicine, healthcare, and daily life. We have transitioned from a field dominated by laser use and therapeutic interventions, to a highly differentiated array of diagnostic and lower-energy therapeutic uses of optics. This field benefits enormously by massive numbers of discoveries in optoelectronic devices that reduce size and costs as components of biomedical systems, and this is paralleled by biomedical applications that have found use for these tools. At this time in the history of our field, it is critical to reexamine the current and future directions where biomedical optics and photonics innovations are having the largest impact, and where they will go in the future. It is with this goal that I wish to give some perspective on this transition of the field and the revision of the scope of the *Journal of Biomedical Optics*.

It is a constant editorial challenge to ensure that we are presenting what our editorial board, SPIE BiOS society members, and our readers are looking for. Our field has grown from the journal being the only one in the field with a small number of papers per issue, to the situation where there are numerous competing journals and a nearly unlimited number of papers in the field. It is imperative to periodically reassess our publication mandate within this ongoing expansion, using the experienced guidance of the editorial board. There are two major observations offered below which together help make the conclusions of the revised scope.

A first observation is that our goal should be to shape the field of biomedical optics by publishing studies that are clearly contextualized as to the hypothesis, discovery, or technical advance, and that this needs to be presented with a comprehensive understanding of the field in which the manuscript is written. In a world where all fields of study are drowning in research publications, it is even more critical to ensure that what gets published in JBO is done so by authors who understand the history and landscape of ‘what is known’ and even more importantly ‘what is not known’ in the field of their manuscript. Indeed, the exponential expansion of journals and published papers today would suggest that all scientific disciplines will continue to be awash in observational studies or papers without the appropriate context. This trend will only increase, and while the search tools to find high-quality studies are getting better, the journals that seek to publish the most impactful state-of-the-art will be the lasting independent markers of quality. This is especially important for community-based journals, such as JBO.

A second observation is that the number of biomedical studies that *use* optical devices for biomedical observations has vastly outpaced the number of studies that report optical innovations or discovery. This is one measure of success in a field that has wide penetration into all aspects of medicine, biology, and society. However, we should not confuse the *use of devices*, with *impactful research*. The core of the SPIE JBO community has always been advancing physics and engineering of biomedical optical innovations, involving nearly equal parts of:

1) advances in optics & photonics devices or systems,
2) computational tools & analysis methods,
3) light–tissue interaction theory and tissue phantoms,
4) initial device/system testing in biology, medicine, or healthcare.

These are at the core of our advances and the biomedical optics component of the SPIE society.

The journal must make difficult choices about what we publish in biomedical optics, with the balance shifted towards excellence in technical innovation and discovery rather than biomedical utility and practice. The editorial board recognizes this phenomenon in our own research circles, and we recognize that seeking to publish innovation and discovery that advance capabilities must outweigh publishing scientific observations that merely document measurements.

The numbers show that technical innovations receive higher downloads and citations than biologically driven innovations, and this is in part because of the fact that biologically driven innovations have many more venues for publication, and the best of these latter papers do not get offered to our journal. In every field of medicine and healthcare there are hundreds of journals that publish relevant papers, and newly emerged systems to image, sense, or measure them are part of their mandate. While we want to encourage and apply the use of optical devices in medicine and via commercialization, if these scholarly activities have impact, they should wind up penetrating into the medical field via medical journals that represent them. For the good of the field, we should be actively advocating for the best biomedical papers to be published in the best biomedical journals, and similarly the best technical papers should be published in the best technical journals. JBO has always represented the edge of both fields, but with a center of gravity that places it as a technical journal primarily.

Technical innovations that have biomedical utility as their goal are well placed in our journal and in the SPIE society. It is with this vision that a slightly revised scope for the journal has been approved by the editorial board. This change is a necessary reassessment of our compass, to tack our trajectory towards the best of our field, and one that will ensure the longevity of the journal and the shape of our field.

In summary, the board of editors and the editorial staff of the *Journal of Biomedical Optics* have approved the following recommendation of a revised scope:

## Scope

The *Journal of Biomedical Optics* (JBO) is an open access journal that publishes peer-reviewed papers on innovations in optical systems and techniques that will improve health care and lead to discoveries in biomedical research. Growth in the capabilities of biomedical optical technology has fueled new areas of contrast, resolution, and spectral capacity in imaging and sensing, which have enabled widespread applications throughout biology and medicine. Topics suitable for JBO include significant in-depth studies of:

• fundamentally new discoveries in biomedical optical devices for imaging or sensing using: optical spectroscopy, near infrared spectroscopy, photoacoustics, microscopy, optical coherence, tomography, fluorescence, phosphorescence, elastography, hyperspectral, using features unique to optics such as spatial, spectral, temporal, interference, polarization, or quantum nature;
• increasing knowledge of light–tissue interaction, through theoretical transport models such as Monte Carlo, diffusion, electromagnetic and empirical methods, and through physical methods to model light–tissue interactions such as tissue-simulating phantoms;
• computational advances in optical image and signal processing, and image reconstruction, including new methods in machine learning and artificial intelligence that improve insight into the utility, detection, or performance value;
• novel medical optical systems used in definitive animal or human studies or clinical trial testing that can impact the field by their design or the optical innovation, including discoveries and technical advances in optics emerging from mobile, remote, wearable, or implantable technologies that can improve health and wellness;
• discoveries in photonics, nanophotonics, plasmonics, biosensors, and optical reporters that have direct relevance to biomedical needs or utility;
• hybrid imaging or interventional systems where optics are combined with other tools such as ultrasound, x-rays, magnetic resonance, molecular sensing, or electromagnetics.

